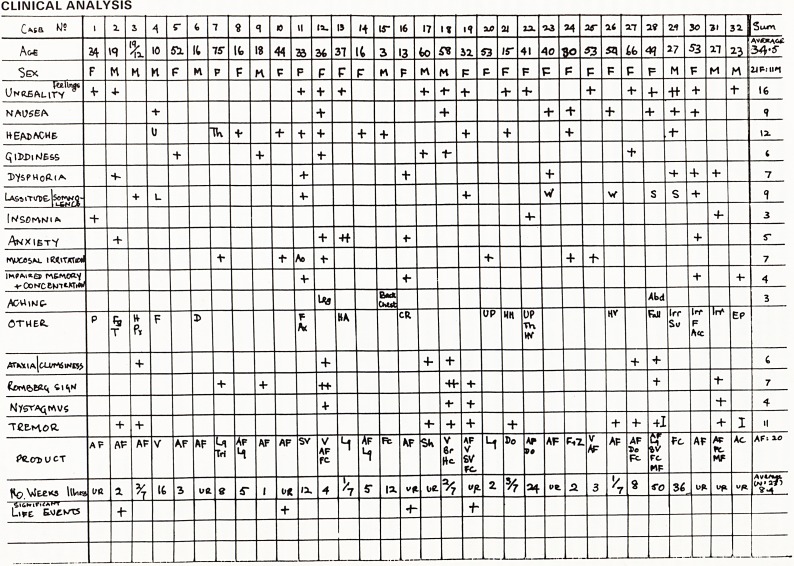# Is There an Air Freshener Syndrome?

**Published:** 1985-01

**Authors:** R. H. Lawson

**Affiliations:** General Practitioner, Congresbury

## Abstract

Thirty two cases are described where the presence of a cluster of neuropsychological symptoms and signs, including unreality feelings, headache, nausea, lassitude, ataxia and tremor, appear to be related to the presence of Air Freshener products. The possible significance of this observation is discussed.


### Is there an Air Freshener Syndrome?

**R. H. Lawson, M.B., B.S., M.R.C.Psych.**
General Practitioner, Congresbury

### SUMMARY

Thirty two cases are described where the presence of a cluster of neuropsychological symptoms and signs, including unreality feelings, headache, nausea, lassitude, ataxia and tremor, appear to be related to the presence of Air Freshener products. The possible significance of this observation is discussed.

### INTRODUCTION

Five years ago, a 34 year old woman presented in the surgery with unreality feelings, palpitations and insomnia. I was unable to find any cause for anxiety, either in her life or in her personality. The following week, she returned and said the Clobazam prescribed for her made her sleepy and did nothing to relieve her symptoms, but that she had noticed that she became worse every time she entered the bathroom. She had removed a 'Stick-Up' Air Freshener from the bathroom, and her symptoms had resolved.

Since that time, I have made it routine to enquire about exposure to 'Freshener' products in patients who complain of neuro-psychological symptoms in the absence of diagnosable, organic or psychiatric syndromes.

### METHOD

These cases were found during routine general practice surgeries in a partnership comprising 4000 patients in a rural area. Socially, the population is predominantly middle-class with young families. The cases were collected over four years.

### SYMPTOMATOLOGY

One of the commonest complaints was the 'muzzy' head. Attempts to get the patient to clarify this symptom are often unrewarding, although 'floating feeling' is frequently advanced as an alternative. However, the patient readily admits on direct questioning to feeling 'here but not here' or 'distant from the things happening around you' and this response was recorded as a symptom of 'unreality feelings'. This state is possibly a mild form of the rarer depersonalisation and derealisation states.

The other symptoms are self-explanatory. In order to avoid leading the patient, when they had completed their spontaenous complaints, they were asked whether there was 'anything else wrong?' before actually embarking on specific direct questioning as to the presence of headache, nausea, vertigo, depersonalisation, tremor, weepiness or tiredness.

Next a brief and informal assessment was made of personality and social situation of the patient, searching for possible pressures and/or causes of a neurosis. It is, of course, nearly always possible to find psychological stresses and strains when sought, but the overall impression was of a non-neurotic 'feel' in those cases which improved after removing the fresheners.

### SIGNS

Examination was limited to brief tests of cerebellar function, covering hand tremor, Romberg's testing (unsteadiness refers to the shoulders moving about 2 cm from the mid-point), heel/toe testing, nystagmus on lateral deviation, and the nose/finger test.

### FRESHENERS

The scent of fresheners is usually apparent when the patient enters the consulting room, or immediately on entering the patient's house (unless, of course, the physician's olfactory nerve is already accommodated through his own use of them). Enquiry on exposure to air fresheners covered each product in turn, as patients will often deny or be unaware of their presence.

A product list is run through mentioning products by name to stimulate memory: 'Air Fresheners – Stick Ups – Airbals – Glade – Haze – Shake'n'vac – Shield or Zest soap – Morning Fresh washing up liquid – Bold 3 – Comfort – Lenor – Sainsbury's fabric conditioner – hair lacquer; in fact anything that says Freshener on it?'

The presence of hair lacquer can be detected by the steadying hand on the head while testing for nystagmus, while a more subtle scent of fresheners on the clothes may be detected during otoscopy.

If a freshener is located in the patient's environment, he or she is aked to remove it: solid block room deodorants are banished to the garden shed, a ban is imposed on sprays and the case of 'freshened' fabric conditioners, it is requested that the clothes

### CLINICAL ANALYSIS

| Case No | 1 | 2 | 3 | 4 | 5 | 6 | 7 | 8 | 9 | 10 | 11 | 12 | 13 | 14 | 15 | 16 | 17 | 18 | 19 | 20 | 21 | 22 | 23 | 24 | 25 | 26 | 27 | 28 | 29 | 30 | 31 | 32 | Sum |
|---|---|---|---|---|---|---|---|---|---|---|---|---|---|---|---|---|---|---|---|---|---|---|---|---|---|---|---|---|---|---|---|---|---|
| Age | 34 | 19 | 19/12 | 10 | 52 | 16 | 75 | 16 | 18 | 44 | 33 | 36 | 37 | 16 | 3 | 13 | 60 | 58 | 32 | 53 | 15 | 41 | 40 | 30 | 53 | 59 | 66 | 49 | 27 | 53 | 27 | 23 | AVERAGE 34·5 |
| Sex | F | M | M | M | F | M | P | F | M | F | F | F | F | F | M | F | M | M | F | F | F | F | F | F | F | F | F | F | M | F | M | M | 21F:11M |
| Unreality Feelings | + | + | | | | | | | | | + | + | + | | | | + | + | + | | + | + | | | + | | + | + | ++ | + | | + | 16 |
| Nausea | | | | + | | | | | | | | + | | | | | | + | | | | | + | + | | + | | + | + | + | | | 9 |
| Headache | | | | U | | | Th | + | | + | + | + | | + | + | | | | + | | + | | | + | | | | | + | | | | 12 |
| Giddiness | | | | | + | | | | + | | | + | | | | | + | + | | | | | | | | | + | | | | | | 6 |
| Dysphoria | | + | | | | | | | | | + | | | | | + | | | | | | | + | | | | | | + | + | + | | 7 |
| Lassitude \| Somnolence | | | + | L | | | | | | | + | | | | | | | | + | | | | W | | | W | | S | S | + | | | 9 |
| Insomnia | + | | | | | | | | | | | | | | | | | | | | | + | | | | | | | | | + | | 3 |
| Anxiety | | + | | | | | | | | | | + | ++ | | | + | | | | | | | | | | | | | | + | | | 5 |
| Mucosal irritation | | | | | | | + | | | + | Ao | + | | | | | | | | + | | | | + | + | | | | | | | | 7 |
| Impaired memory + concentration | | | | | | | | | | | + | | | | | + | | | | | | | | | | | | | | + | | + | 4 |
| Aching | | | | | | | | | | | | Leg | | | Back Chest | | | | | | | | | | | | | Abd | | | | | 3 |
| Other | P | Fg T | H Px | F | | D | | | | | F Ax | | HA | | | CR | | | | UP | HH | UP Th HV | | | | HV | | Fall | Irr Su | Irr F Acc | Irr | EP | |
| Ataxia \| Clumsiness | | | + | | | | | | | | | + | | | | | + | + | | | | | | | | | + | + | | | | | 6 |
| Romberg sign | | | | | | | + | | + | | | ++ | | | | | | ++ | + | | | | | | | | | + | | | + | | 7 |
| Nystagmus | | | | | | | | | | | | + | | | | | | + | + | | | | | | | | | | | | + | | 4 |
| Tremor | | + | + | | | | | | | | | | | | | | + | + | + | | + | | | | | + | + | +I | | | + | I | 11 |
| Product | AF | AF | AF | V | AF | AF | Lq Tri | AF Lq | AF | AF | SV | V AF FC | Lq | AF Lq | Fc | AF | Sh | V Br Hc | AF V SV FC | Lq | Do | AF Do | AF | F+Z | V AF | AF | AF Do Fc | AF Lq SV Fc MF | Fc | AF | AF Fc MF | AC | AF: 20 |
| No. Weeks Illness | UR | 2 | 2/7 | 16 | 3 | UR | 8 | 5 | 1 | UR | 12 | 4 | 1/7 | 5 | 12 | UR | UR | 2/7 | UR | 2 | 3/7 | 24 | UR | 2 | 3 | 1/7 | 8 | 50 | 36 | UR | UR | UR | Average (N=23) 8·4 |
| Significant Life Events | | + | | | | | | | | + | | | | | | + | | | + | | | | | | | | | | | | | | |

and bed-clothes are rinsed again. This is a lot to ask of a person but complete compliance is necessary if improvement is to take place. The patient is then recalled at weekly intervals. Improvement will have usually come about within one week if it is to occur at all; but in the case of fabric conditioners, especially if compliance is low, it may take 2 weeks or more.

**ANALYSIS OF DATA**

In decreasing order of frequency, the key symptoms are as follows:

Unreality feelings (16)
Headache (12)
Tremor (4)
Nausea (9)
Lassitude and Somnolence (9)
Depression and weepiness (7)
Mucosal irritation (7) including nose, eyes, throat, chest and Urethra
Giddiness and Ataxia (6 each)
Anxiety (5)
Impaired memory and concentration (4)

There were three presentations each of: irritability, fainting, insomnia and muscular aching.

### DISCUSSION

There are, of course, two possible explanations for these observations.

First, they may be quite spurious. Improvement may be due entirely to placebo effect, a response to the physician's suggestion of improvement, or out of a desire to please. This is difficult to disprove but if similar results have been, or might be, found by independent physicians, it would become less likely.

On the other hand, there might be a cause effect relationship between the 'freshener' odourants and the symptom cluster described. This is quite conceivable in theory. The sense of smell is intimately connected with the emotions.[1,2] In the animal world, odourants are used as a chemical communication system which governs many aspects of their behaviour.[3] Anatomically the olfactory nerve projects onto the limbic system (previously known as the rhinencephalon) which is now known to mediate emotions. It also projects directly onto the amygdaloid complex which as well as being associated with the release of aggressive behaviour, is also linked via the caudate nucleus to the basal ganglia which control muscle tone.

The fresheners themselves, consist of a number of natural, and synthetic perfumes. The ratio of synthetic to natural is greater in the fresheners than in traditional perfumes. They act by stimulating the olfactory nerve more strongly than undesired odours. Hypothetically, this stimulation might irradiate onto the limbic system to produce emotional stimulation (tearfulness and/or irritability) followed by emotional blunting (causing 'unreality' feelings). The olfactory irradiation to the amygdala might mediate the tremor and instability that is found in these cases and the irradiation onto the hypothalamus might produce the nausea.

KEY

Symptoms

Abd: Abdominal ache
Acc: Accomodation difficulty
Ao: Anosmia
Ax: Anorexia
CR: Compulsive Rituals
D: Delerium
EP: ? Epileptic fits
F: Fainting (3)
Fg: Fugue
Fall: Fall Sensation
H: Hallucination
HA: Hemi anaesthesia
HH: "Heavy hands"
HV: Hyper ventilation
I: Intention tremor
Irr.: Irritability (3)
L: Lassitude
P: Palpitations
Px: Perplexity
S: Somnolence
Su: Suicidal impulse
T: Twitching
Th: Throbbing
U: Unilateral headache
UP: Unilateral Paraesthesia
UR: Unrecorded
W: Weakness

Products

| | |
|---|---|
| AF: Air Freshener (solid block room deodorant) | 20 |
| Br: Brut | 1 |
| Do: Personal deodorants | 3 |
| F: 'Fresh' soap | 1 |
| FC: Perfumed Fabric Conditioners | 7 |
| HC: Herbicide | 1 |
| MF: Morning Fresh Washing Up Liquid | 2 |
| Lq: Hair Lacquer | 6 |
| Sh: 'Shield' soap | 1 |
| SV: Shake'n'Vac Carpet Cleaner | 3 |
| Tri: Triazolam | 1 |
| V: Vapona Fly Killing block | 5 |
| Z: 'Zest' soap | 1 |

In addition to the perfumes themselves, other chemicals are likely to be present, and I am indebted to Mr. Cooper of the City of Bristol Environmental Health Department for the following review:

*Fluorocarbons* are almost certain to be the propellants in any aerosol products. There is a popular belief that these are safe under all conditions of exposure, but this is not necessarily the case. The volatile compounds are known to possess narcotic properties. If inhaled at 5% v/v concentration, dichlorodifluoromethane ($CCl_2F_2$), for instance, induces dizziness. Admittedly, 5% is quite a gross concentration, unlikely to be approached without deliberate capture and inhalation, but lower concentration of this group of products cannot be exonerated from any effect.

*Perfumes* may incorporate a very wide range of chemicals, including hydrocarbons, aldehydes, ketones, ethers and miscellaneous compounds. The use of solvents which, in moderate to high concentrations may have intoxicating effects, cannot be ruled out, although, again, concentrations occurring through domestic use are probably unlikely to reach levels regarded as toxic.

*Alcohols* are liable to be present in some of the products mentioned. Although most of this group of compounds are not regarded as highly hazardous inhalation, the irritant and intoxication properties of some are well recognised.

*Ketones* These compounds present a moderate health hazard in industrial concentrations and possess narcotic properties when inhaled in high concentrations. The vapours are, however, irritating to the eyes and respiratory system in concentrations much lower than those that produce a narcotic effect: They may occur as solvents in cosmetics, perfumes and lacquers.

*Aldehydes* are associated with the production of perfumes and essences and have a tendency to cause irritation of the skin, eyes and respiratory system. In certain instances, their physiological action is to provide a hypnotic effect.

Although these observations by no means prove that Air Fresheners can constitute a health risk, they do demand that this matter be studied further since the substances are already widespread throughout our environment, and it is possible that some sufferers may be receiving inappropriate psychiatric treatment.

There are three possible approaches to verification or rejection of these findings.

(1) Further empirical clinical work in a general practice setting. This work would be best carried out by a physician sceptical of the suggestions in this paper.

(2) Controlled exposure of both sensitive and unaffected subjects to inhaled fresheners while performing neuro-psychological tests.

There is an ethical problems here in exposing people again to an agent thought to do them harm, though most of the subjects in the present series have said that they would be willing to take part in such an experiment. However, the question of suggestibility arises again since the subjects will be aware when fresheners are introduced, and this could be said to invalidate the experiment. On the other hand if the olfactory nerve were to be anaesthetized the mode of action of the fresheners might be blocked also.

(3) Social surveys could be carried out with a present state questionnaire presented to hundreds of people, followed by a questionnaire related to their exposure to fresheners, followed by a further present state questionnaire afer removal of the fresheners.

### ACKNOWLEDGEMENTS

Many thanks to Mrs. Jean Woolner for her patient work with the typewriter.

### REFERENCES

1. FISCHER R.—Gustatory, Behavioural & Pharmacological manifestation of chemoreception in man, in Gustation & Olfaction International Symposium, Geneva. June 1970 eds. G. Ohlof & A. E. Thomas, Academic Press, London and New York.
2. DODD. G., VAN TOLLER S., Perfumer and Flavorist 1983 Vol. 8 No. 4.
3. Olfaction in mammals. Proc. 45th Symposium of the Zoological Society of London. D. M. Stoddart ed., Academic Press, London and New York (1980).

## Figures and Tables

**Figure f1:**